# Challenges in Poultry Production Systems and Nutritional Interventions

**DOI:** 10.3390/ani15040530

**Published:** 2025-02-13

**Authors:** Janghan Choi

**Affiliations:** Department of Animal and Food Sciences, Texas Tech University, Lubbock, TX 79409, USA; janghan.choi@ttu.edu

Chicken is the most produced and consumed meat in the world [1]. Poultry production yields affordable and highly nutritious (e.g., protein, vitamins, irons, omega 3, etc.) meat and eggs all over the world. There was a great achievement in poultry production that increased the meat and egg yields, production efficiency, which was resistant against specific challenges over recent decades [2,3]. However, there are still many challenges that can negatively impact productivity, efficiency, food safety, and animal welfare in poultry production [4]. Challenges in poultry production, such as feed costs, parasitic and bacterial infections, heat stress, muscle myopathies, mycotoxin contamination, and issues related to rearing conditions and stocking density, have prompted extensive research into diverse nutritional and systemic interventions to address these problems.

This Special Issue collects valuable research articles and reviews describing the current challenges in poultry production systems, and nutritional and systemic interventions to cope with these issues. Parasitic and bacterial infections are some of the major issues that negatively influence production, welfare, and food safety issues in poultry production, and the withdrawal of anticoccidial and antibacterial drugs has aggravated the negative impacts of the challenges in poultry production [5,6]. *Eimeria* infection alone, or with *Clostridium perfringens* (e.g., necrotic enteritis) cause tremendous economic losses in poultry production [7,8]. In this Special Issue, Choi et al. [9] demonstrated that different *Eimeria* infection doses negatively impact productivity and efficiency in broiler chickens raised in floor pens by negatively impacting their feed intake, nutrient digestibility, gut microbiota, and foot pad dermatitis. Furthermore, this study provided information on *Eimeria* infection models (e.g., infection doses and their responses) for broilers in the floor pen condition. Most of the previous *Eimeria* research in broiler chickens was conducted in cage studies, while most of the broilers are normally raised in the floor pen condition in the US [10]. Sharma et al. [10] showed that inflammatory response in *Eimeria* infection and necrotic enteritis could disrupt the growth and development of bone via regulating RANKL-RANK pathway in chickens. Zhang et al. [11] demonstrated that the supplementation of 1000 mg/kg tannic acid (plant extract), which is known to have antimicrobial, antioxidant, and anti-inflammatory effects [4,12,13], improved gut integrity, attenuated the inflammatory response, and enhanced antioxidant capacity in broiler chickens under the necrotic enteritis challenge. Moreover, Yang et al. [14] showed that microencapsulated sodium butyrate (e.g., an organic acid) enhanced immune status, intestinal morphology, and volatile fatty acid production, mainly by beneficially affecting gut microbiota in broiler chickens infected with *Clostridium perfringens*. Zhi et al. [15] showed that the supplementation of sea-buckthorn flavonoids (e.g., plant extracts) improved growth performance, systemic immune responses, and gut barrier integrity in broiler chickens challenged with bacterial lipopolysaccharides. Our Special Issue highlighted the detrimental effects of parasitic and bacterial infections on broiler chickens, while presenting variety of nutritional strategies to mitigate these challenges.

Improving productivity and efficiency by nutritional interventions is also important in general conditions in poultry production. Phosphorus, the third-most expensive nutrient in the poultry diet, plays important roles in regulating energy metabolism, cell homeostasis, bone growth, gut health, and overall performance in chickens [16,17]. Decreasing phosphorous levels would be beneficial to decrease feed costs and to reduce phosphorus excretion to the environment, while it can decrease the productivity and efficiency of the poultry production [18]. The supplementation of phytase could increase the utilization of phosphorus in phosphorus-deficient diets. In this Special Issue, Yu et al. [19] demonstrated that the supplementation of an *Escherichia coli*-derived 6-phytase improved growth performance and bone health in broilers fed phosphorus-deficient diets by improving the utilization of phosphorus. These results suggest that the reduction of phosphorous with the addition of the phytase would not negatively impact the growth of broilers via enhancing phosphorus utilization. Corn–soybean meal diets have high contents of non-starch polysaccharide (NSP) including arabinoxylan, β-glucan, cellulose, xylose, mannose, and pectin, which can lead to poor productivity and efficiency by decreasing feed intake and compromising nutrient digestion and absorption, mainly through increasing the digesta viscosity in the gastrointestinal tract of chickens [20,21]. Shuaib et al. [22] showed that the addition of a β-mannanase with the inclusion of 10% soyhull, which is rich in NSP, increased the growth performance of laying hens during the late peak production without affecting the egg production and quality. Their study demonstrated that supplementation of phytase and β-mannanase could improve the nutrient utilization of poultry.

Rearing temperature and environments are crucial factors, and as important as the nutritional and genetic factors in poultry production. In the global warming era, the temperature in the poultry house and in the feed mill is increasing. Poultry is sensitive to heat stress, which can negatively impact animal welfare, growth performance and egg production, and quality of chickens [23]. In this Special Issue, Sumanu et al. [24] demonstrated that the supplementation of a probiotic (*Saccharomyces cerevisiae*) and ascorbic acid improved growth performance, intestinal morphology, and antioxidant capacity heat stress in broilers subjected to heat stress. The incidence and severity of mycotoxins contamination in the ingredients and feeds are increasing with the global warming [25]. Lee et al. [26] showed that the inclusion of the two toxin binders, namely (1) clay minerals (85% bentonite and 12% clinoptilolite) with 3% charcoal and (2) clay minerals (66.1% aluminosilicates), with natural components (0.8% artichoke and rosemary plant extracts, 7% yeast extract, 0.5% beta-glucans, and 25.6% carriers), attenuated the negative impacts of feeding diets contaminated by ochratoxin A (a highly toxic mycotoxin produced by several fungi, including the Aspergillus and Penicillium genera) in broiler breeders. Low rearing temperatures (e.g., cold stress) could influence the physiology of chickens [27]. Kim et al. [28] demonstrated that low rearing temperatures impaired the antioxidant system and modulated lipid metabolism in laying hens without affecting egg production and quality in layers. Al-Baadani et al. [29] and Kang et al. [30] demonstrated that environmental factors could affect animal welfare, productivity, efficiency, and systemic health in chickens. Al-Baadani et al. [29] demonstrated that high stocking density could compromise productivity, efficiency, meat yield, and gut health, and the supplementation of gum Arabic and commercial prebiotics improved growth performance, production efficiency, and gut health of broiler chickens, potentially by beneficially modulating gut microbiota. Kang et al. [30] demonstrated that stimulated voluntary activities by placing enrichment huts in the light enriched broiler house improved animal welfare and systemic health in fast growing modern broilers. Our Special Issue provides variable strategies to cope with diverse environmental strategies.

Taken together, diverse challenging conditions in poultry production and effective nutritional and systemic coping strategies were researched and discussed in this Special Issue. There is still not a single bullet to resolve the current challenges in poultry production yet. Continuous studies with novel ideas are required to understand the challenges, and to elucidate the coping strategies in poultry production.
